# Supplementary material for: Stochasticity contributes to explaining minority and majority MOMP during apoptosis
Source: Cell Death Dis. 2025 Dec 19;16(1):893. doi: 10.1038/s41419-025-08258-9 (PMC12717247; doi:10.1038/s41419-025-08258-9)
Supplement: Supplementary file 1 — Supplemental Material [file 41419_2025_8258_MOESM1_ESM.docx]

**SUPPLEMENTAL INFORMATION for**

**Stochasticity contributes to explaining minority and majority MOMP during apoptosis**

Jenny Geiger^1^, Fabian Klötzer^1^, Nadine Pollak^1,2^, Gavin Fullstone^1,2,3,*^, Markus Rehm^1,2,*^

^1^ Institute of Cell Biology and Immunology, University of Stuttgart, 70569 Stuttgart, Germany.

^2^ Stuttgart Research Center Systems Biology, University of Stuttgart, 70569 Stuttgart, Germany.

^3^ School of Medicine, Pharmacy and Biomedical Sciences, University of Portsmouth, PO1 2DT Portsmouth, United Kingdom.

*To whom correspondence should be addressed.

| Dr Gavin Fullstone  School of Medicine, Pharmacy and Biomedical Sciences  University of Portsmouth  St Michael’s Building, White Swan Road, Portsmouth, PO1 2DT, Portsmouth  Phone: +44 23 9284 3546  E-mail: [gavin.fullstone@port.ac.uk](mailto:gavin.fullstone@port.ac.uk)  ORCID: 0000-0001-5246-3443 | Prof Dr Markus Rehm  Institute of Cell Biology and Immunology  University of Stuttgart  Allmandring 31, 70569 Stuttgart, Germany  Phone: +49 711 685-66987  E-mail: [markus.morrison@izi.uni-stuttgart.de](mailto:markus.morrison@izi.uni-stuttgart.de)  ORCID: 0000-0001-6149-9261 |
| --- | --- |

**Key Words:** mitochondrial outer membrane permeabilization (MOMP), particle-based modelling, cell death, apoptosis

**Supplemental materials and methods**

**Defining simulation parameters for particle-based modeling of MCL-1 translocation**

Various parameters, including protein concentration, cellular, and mitochondrial characteristics, needed to be quantified and incorporated into the particle-based model. These parameters are described in the following sections.

The implementation of protein concentration as particle numbers was achieved by deriving median MCL-1 concentrations from gated flow cytometry data using a modified version of MAPIT (1). The median MCL-1 concentration was determined to be 101 nM. The BAK concentration in NCI-H460 cells was measured at 1002 nM via comparative densitometry analysis of Western blots. These concentrations were converted into absolute molecule counts using the following formula:

$N= c*V_{c}* N_{A}$ (1)

where $N$ is particle number, $c$ is the concentration $\left[ \frac{\mathrm{mol}}{L} \right]$, $V_{c}$ is the cell volume $\left[ L \right]$ and $N_{A}$ is Avogadro constant. For this conversion, an in-house measured cell volume of 3.917*10^-12^ L (G_1_ phase) of NCI-H460 cells was used. The calculated particle numbers for the proteins incorporated into the model are provided in **Supp. Fig. 1A**.

Protein concentrations in HCT116 cells were also quantified using comparative densitometry of Western blots. These relative concentrations were converted into absolute molecule numbers using equation [1] and a measured average cell volume of 2.441*10⁻¹² L for HCT116 cells. The resulting particle counts for proteins incorporated into the model are shown in **Sup. Fig. 5 A**.

The diffusion coefficient for soluble proteins is required by FaST to simulate the random walk (2). Size dependent cellular diffusion coefficients, $D$, were estimated using the Stokes-Einstein formula and the measured cellular diffusion rate of GFP, as described previously (3):

$D=D_{\mathrm{GFP}}*\left( \frac{m_{\mathrm{GFP}}}{m_{MCL-1}} \right)^{\frac{1}{3}}=24 \frac{{\mu m}^{2}}{s}*\left( \frac{27 kDa}{37 kDa} \right)^{\frac{1}{3}}=21.607 \frac{{\mu m}^{2}}{s}$ (2)

where $m_{MCL-1}$ represents the molecular weight of MCL-1, $m_{\mathrm{GFP}}$ is the molecular weight of GFP and $D_{\mathrm{GFP}}$ is the measured diffusion rate of GFP in cells (4).

A diffusion coefficient of 21.607 µm^2^s^-1^ was assigned for MCL-1 and remained unchanged across different model conditions. The diffusion coefficient for a BCL-2 family protein, BCL-XL, bound to lipid bilayers in giant unilamellar vesicles (GUVs) was previously experimentally determined as 4.8 µm^2^s^-1^ (5), this value was used as the diffusion coefficient for all BCL-2 family proteins on membranes based on the assumption that molecular weight will have a lower impact on diffusion within a membrane according to the Saffman-Delbrück theory (6).

**Specification of the *in-silico* cell geometry based on high-resolution 3D imaging**

In-house measurements of NCI-H460 cells size from 3D-imaging data were used to construct a representative three-dimensional simplified model cell containing mitochondria. In brief, the acquisition protocol was as follows: untreated cells were stained with MitoTracker^TM^ (MitoTracker^TM^ Red CMXRos (M7512, Thermo Fisher scientific corporation)) and microscopy images were acquired using Airyscan Joint Deconvolution in Superresolution mode. The whole cell volume was measured using the MCL-1 signal across the whole cell. Mitochondrial network parameters (mitochondrial number, mitochondrion surface area and volume) were extracted using the Mitochondria Analyzer pipeline in ImageJ and are shown in **Supp. Fig. 1B**. The same procedure was followed to obtain whole cell volume and mitochondrial parameter measurements in HCT116 cells (**Supp. Fig. 5 B**).

This data was used to determine the dimensions of the model cell to ensure an equivalent volume. The mitochondria within the model were assigned to a rectangular shape with the same number, surface area and volume as the analysed mitochondria. The final model parameters for the mitochondria and the cell for the model cell environment are summarized in **Supp. Fig. 1C** for an NCI-H460 model cell and in **Supp. Fig. 5 C** for a HCT116 model cell.

**Determination of kinetic parameters for MCL-1 translocation**

To implement translocation of MCL-1, fluorescence recovery after photobleaching (FRAP) was combined with steady state measurements of MCL-1 localisation to capture both dynamics (from FRAP) and distributions in NCI-H460 cells. FRAP was performed by bleaching MCL-1 on stage, which was endogenously fluorescence tagged via mScarlet knock-in. After the bleaching, the fluorescence recovery within the bleached area was measured, to estimate the translocation kinetics of MCL-1 (**Supp. Fig. 2D**). The FRAP profile was used to obtain the fluorescence recovery rate of 0.00452 s^-1^ using non-linear one-phase association regression analysis (**Supp. Fig. 2E**). In-house imaging data of immunofluorescence-stained MCL-1 combined with markers for mitochondria and nuclei was used to quantify steady state MCL-1 concentrations in the cytoplasm (MCL-1_Cyto_) and mitochondria (MCL-1_Mito_) revealing that the [MCL-1_Cyto_]:[MCL-1_Mito_] ratio is 0.7923 (**Fig. 1C**). From these values, a fitting function was derived by integrating the fluorescence recovery rate with the [MCL-1_Cyto_]:[MCL-1_Mito_] ratio, resulting in the function:

${MCL-1}_{\mathrm{Mito}}\left[ \mathrm{particles} \right]*\left( 1-e^{k*t} \right)=28671*\left( 1-e^{-0.00452s^{-1}*t} \right)$ (3)

Where 28671 represents the absolute amount of MCL-1 particles on the mitochondria to give the expected [MCL-1_Cyto_]:[MCL-1_Mito_] ratio of 0.7923. This data was used to estimate k_on_ and k_off_ values of MCL-1 translocation dynamics. A least-squares global parameter estimation was conducted using a basic ODE model:

$\frac{d\left[ {MCL-1}_{\mathrm{Cyto}} \right]}{\mathrm{dt}}=k_{\mathrm{off}}\left[ {MCL-1}_{\mathrm{Mito}} \right]-k_{\mathrm{on}}\left[ {MCL-1}_{\mathrm{Cyto}} \right]$ (4)

$\frac{d\left[ {MCL-1}_{\mathrm{Mito}} \right]}{\mathrm{dt}}=k_{\mathrm{on}}\left[ {MCL-1}_{\mathrm{Cyto}} \right]-k_{\mathrm{off}}\left[ {MCL-1}_{\mathrm{Mito}} \right]$ (5)

Estimated upper and lower bounds for k_on_ and k_off_ were set between 0.01 s^-1^ and 0.00001 s^-1^. The values with the lowest estimation cost were selected for further analysis, resulting in k_on_ 0.00054367 s^-1^ and k_off_ 0.0039562 s^-1^. These values were validated by reintroducing them into the basic ODE model, which successfully reproduced the experimentally obtained [MCL-1_Cyto_]:[MCL-1_Mito_] ratio of 0.7923, with the model yielding a ratio of 0.793. These estimated k_on_ and k_off_ values were integrated into the particle-based model by calculating probability values. For translocation of MCL-1_Cyto_ to the mitochondria, first the collision rate of MCL-1 colliding with mitochondria was determined by simulating MCL-1 diffusion in the cytoplasm and calculating collisions with mitochondria. Collisions refers to when a molecule of MCL-1_Cyto_ crosses a reflective boundary of a mitochondrion. The collision rate, of 195.169862 s^-1^ and the k_on_ of 0.00054367 s^-1^ estimated above were used to define the binding probability:

$\mathrm{probability}_{\mathrm{binding}}=\frac{k_{\mathrm{on}}}{collision rate}=\frac{0.00054367 s^{-1}}{175.169862 s^{-1}}=3.10367*{10}^{-6}$ (6)

Likewise, the estimated k_off_ value was used to calculate the unbinding probability, describing the MCL-1_Mito_ detachment from the mitochondrial membrane:

$\mathrm{probability}_{\mathrm{unbinding}}=1-e^{-k_{\mathrm{off}}*\Delta t}=1-e^{-0.0039562 s^{-1} * 0.05 s}=1.9779*{10}^{-4}$ (7)

A function was implemented to generate a random number between 0 and 1 to determine if binding upon collision or unbinding occurs after each timestep. In the event that a collision of MCL-1_Cyto_ did not lead to a binding event, the MCL-1_Cyto_ particle was reflected off the mitochondrial surface. In the event of binding, the MCL-1 molecule was assigned as MCL-1_Mito_ and localised at the point of intersection with the mitochondrial surface.

**Parameter estimation for simulating mitochondrial protein–protein interactions**

Previously, we published an ODE-model of BCL-2-family interactions, incorporating a representative of the BH3-only proteins (tBID), an anti-apoptotic protein (BCL-XL) and a pore-forming effector protein (BAX) (7). This model, specifically its reaction network, was used as the basis for the particle-based models of events at individual mitochondria. We, however, made the following changes. Firstly, we used MCL-1 instead of BCL-XL as a representative anti-apoptotic protein on the basis that though functionally similar, we have recently demonstrated experimental capture of MCL-1 localization behavior in cells during the cell cycle (8). This data and the experimental tools generated from this study and ongoing follow up studies allow us to better experimentally define MCL-1 steady state and retrotranslocation dynamics than for BCL-XL. Importantly, key kinetic rates, such as retrotranslocation of BAX and BAK, are consistent between BCL-XL and MCL-1 (9). Secondly, we used BAK instead of BAX as a representative pore-forming effector protein. Whilst functionally similar, BAX is predominantly found in the cytoplasm but migrates to the mitochondria upon apoptotic stress whereas BAK is predominantly located at the mitochondrial membrane. The use of BAK allowed us to neglect the effect of dynamic exchange of BAX between mitochondria and the cytoplasm, which is highly computationally challenging, and focus on capturing snapshots of MOMP-sensitivity. Importantly, as (retro)translocation times are generally slower than diffusion dynamics, the effect of stochasticity on BAX distributions is expected to be generally higher than for BAK. Therefore, despite these changes, our findings are relevant to the extended network of BCL-2 family interactions.

To parameterize the model, it was necessary to undertake a parameter estimation to obtain a single parameter set that represents both the expected dynamic behavior of BCL-2 family interactions and reproduces quantitative experimental reference data. This section describes the estimation of these parameters.

**Mitochondrial Model Parameter Estimation: Definition of Reference Data**

To undertake the parameter estimation, 2 sets of experimental reference data were extracted from the publication of Subburaj and colleagues (Fig. 3G and 4D from (10)) using ImageJ. The first data set describes the formation of BAX oligomers for 1 h in the presence of cBID on supported lipid bilayers (SLBs) mimicking the composition of the mitochondrial membrane (hereafter referred to as Estimation Data 1). The second data set describes the BCL-XL-mediated disruption of pre-formed BAX oligomers after 1 h (hereafter referred to as Estimation Data 2). In both experiments, the readout was percentage occurrence of different BAX oligomers on SLBs (monomers, dimers, trimers, tetramers, pentamers and hexamers). Notably, the experimental setup used for Estimation Data 2 includes a 1 h pre-formation of BAX oligomers by incubation of BAX and cBID with the exact same experimental conditions as used to generate Estimation Data 1. Therefore, a parameter estimation was performed based on 2 sequential simulations representing both Estimation Data 1 and 2. First, a 1 h simulation was performed using the initial conditions shown in **Supp. Fig. 1D**.

At the end of the first simulation (Simulation 1), the cost (Cost 1) was calculated from the end points of the simulation and Estimation Data 1:

$Cost 1= \sum{(model-experiment)}^{2}$ (8)

A detailed explanation of how model data and experimental data were prepared and compared is given in the next section. Thereafter, the end of Simulation 1 was used to initialise Simulation 2 with all species and intermediates from Simulation 1 after 1 h carried over with the addition of 2.5 nM of BCL-XL. Simulation 2 was then performed for 1 h and the cost for Simulation 2 calculated (Cost 2) in the same way as Cost 1 (Equation 8). The total cost for a parameter set was given by:

$Total Cost=Cost 1+Cost 2$ (9)

**Mitochondrial Model Parameter Estimation: Comparing Simulation and Experimental Values**

The experimental data was measured as percentage occurrence of each BAX oligomer on the SLB membrane after 1 h. As no trimers and pentamers were observed experimentally and were explicitly excluded from the model reaction network, the model and experimental levels of monomeric, dimeric, tetrameric and hexameric BAX were used for the cost analysis. For monomers of BAX from simulations, the quantities of aBAX, tBID-bound BAX and BCL-XL-bound BAX were added together to represent all species of BAX expected to be detectable within membranes (Equation 10). For dimers, tetramers and hexamers, the concentration of the equivalent species was taken directly from the model (aBAX2, aBAX4 and aBAX6 respectively). As the experimental data was based on area under the curve measurements from the fitting of six Gaussian curves (representing monomers, dimers, trimers, tetramers, pentamers and hexamers) to a frequency distribution (fluorescence intensity vs number of events) to estimate percentage occurrence of each species, the concentration of BAX multimers from simulations was multiplied by the oligomerisation state (i.e. 2× for dimers, 4× for tetramers). To normalise the simulation data to percentage occurrence, the sum of all BAX species, including oligomers, expected to be detectable within membranes (i.e. all except inactive BAX monomers) multiplied by their respective BAX oligomerisation state was calculated (see Equations 10-13).

$$BAX monomer occurrence \left( \% \right)=$$

$100\%\cdot\frac{\left[ \mathrm{aBAX} \right]+\left[ \mathrm{tBIDBAX} \right]+\left[ \mathrm{tBIDaBAX} \right]+[BCLXLaBAX]}{\left[ \mathrm{aBAX} \right]+2\cdot\left[ aBAX2 \right]+4\cdot\left[ aBAX4 \right]+6\cdot\left[ aBAX6 \right]+\left[ \mathrm{tBIDBAX} \right]+\left[ \mathrm{tBIDaBAX} \right]+[BCLXLaBAX]}$ (10)

$BAX dimer occurrence \left( \% \right)=$ $100\%\cdot\frac{2\cdot\left[ aBAX2 \right]}{\left[ \mathrm{aBAX} \right]+2\cdot\left[ aBAX2 \right]+4\cdot\left[ aBAX4 \right]+6\cdot\left[ aBAX6 \right]+\left[ \mathrm{tBIDBAX} \right]+\left[ \mathrm{tBIDaBAX} \right]+[BCLXLaBAX]}$ (11)

$BAX tetramer occurrence \left( \% \right)=$

$100\%\cdot\frac{4\cdot\left[ aBAX4 \right]}{\left[ \mathrm{aBAX} \right]+2\cdot\left[ aBAX2 \right]+4\cdot\left[ aBAX4 \right]+6\cdot\left[ aBAX6 \right]+\left[ \mathrm{tBIDBAX} \right]+\left[ \mathrm{tBIDaBAX} \right]+[BCLXLaBAX]}$ (12)

$BAX hexamer occurrence \left( \% \right)=$

$100\%\cdot\frac{6\cdot\left[ aBAX6 \right]}{\left[ \mathrm{aBAX} \right]+2\cdot\left[ aBAX2 \right]+4\cdot\left[ aBAX4 \right]+6\cdot\left[ aBAX6 \right]+\left[ \mathrm{tBIDBAX} \right]+\left[ \mathrm{tBIDaBAX} \right]+[BCLXLaBAX]}$ (13)

**Mitochondrial Model Parameter Estimation: Definition of Upper and Lower Bounds**

Model parameters were constrained to biological plausible lower and upper limits similar to our previously developed model (7). For forward reactions, reactions were constrained between 1E3 – 1E6 M^-1^·s^-1^. K_D_ ranges of 1E-10 –1E-6 M was previously determined to be reasonable ranges for BCL-2 family interactions (7). Therefore, reverse reaction rates were set by estimating the forward reaction rates and K_D_s, through the relationship:

$k_{r}=k_{f}\cdot K_{D}$ (14)

First order enzymatic reactions (**Supp. Fig. 1F**), were constrained between 0.1–1E-7 s^-1^ and retrotranslocation rates (**Supp. Fig. 1E**) were constrained between 0.1–1E-5 s^-1^ as performed previously based on experimentally measured retrotranslocation rates (7).

Initial parameter estimations based on these boundaries yielded an unsatisfactory cost and reproduction of the reference data. Moreover, several parameters were frequently located towards the edge of their constraints. It was therefore reasoned that a neglected aspect of using an ODE paradigm for the parameter estimation was the concentrating effect of several reactions occurring exclusively at mitochondrial membranes (a factor intrinsically addressed in our particle-based simulations). Therefore, for second order reactions that occur exclusively at membranes, the upper bounds for forward rates were increased by 100× (reflecting an ~10-fold concentrating effect on both reactants). Reciprocally, the K_D_ lower bounds for these reactions were lowered by 100×. The resulting parameter estimation constraints (see **Supp. Fig. 1D**) led to a vastly improved reproduction of the reference data.

**Mitochondrial Model Parameter Estimation: Implementation of a Global Parameter Estimation**

The parameter estimation was performed in MATLAB (R2020b) using previously established scripts for a global parameter estimation (2). In brief, initial parameter sets were selected within pre-defined parameter ranges by Latin hypercube sampling. For each initial parameter set, a local minima was determined using the MATLAB function fmincon (<https://de.math-works.com/help/optim/ug/fmincon.html>). The parameter estimation was performed in parallel across 12 worker nodes for 72 h, giving a final total of 6153 iterations with local minima. The parameter set with the least global cost were chosen as the best fit parameter set.

**Mitochondrial Model Parameter Estimation: Results**

The global minima from the parameter estimation had a cost of 199.5 and showed reasonable replication of Estimation Data 1 (**Supp. Fig. 2A**) and Estimation Data 2 (**Supp. Fig. 2B**). Full simulation outputs showed a strong effect of the addition of BCL-XL in the rapid de-assembly of BAX oligomers and retrotranslocation of BAX. This strong inhibitory effect of anti-apoptotic BCL-2 family proteins on pore formation and particularly the role played by retrotranslocation was shown by us to be an integral feature of apoptotic signalling to achieve the generally all-or-nothing response of mitochondrial outer membrane permeabilization (7). Based on this and its good reproduction of quantitative reference data, these parameters were then used in the particle-based simulations of mitochondrial pore formation.

**Incorporation of BAX into the model for simulating protein reactions at single-mitochondrion resolution**

BAX was integrated into a reduced pro-apoptotic interactome consisting of MCL-1, tBID, and BAX, modeled at the level of individual mitochondria in a representative NCI-H460 cell. The cellular concentration of BAX (606 nM) was determined via Western blot densitometry. Unlike BAK, BAX required special handling due to its differential cellular localization and dynamic (retro-)translocation between the cytosol and mitochondria. Simulating translocation of BAX from the cytoplasm to the mitochondria and the protein reactions simultaneously in the whole cell were computationally too expensive due to the number of reactants and species to be modelled. Therefore, the model was optimized to simulate MOMP susceptibility on a single mitochondrion resolution as described in the Materials and Methods section.

To implement BAX translocation, we first established a baseline of mitochondrial-bound BAX corresponding to a mito:cyto ratio of 1.1, based on the initial YFP-BAX_Mito_:YFP-BAX_WholeCell_ ratio measured in BAK-deficient DU-145 cells (Figure 5D in [11]; see Equations 15 and 16).

$\mathrm{BAX}_{\mathrm{Cyto}}\left[ \mathrm{particles} \right]=\frac{\mathrm{BAX}_{\mathrm{total}} \left[ \mathrm{particles} \right]}{1.1+1}=\frac{1431538}{2.1}\approx681684 particles$ (15)

$\mathrm{BAX}_{\mathrm{Mito}}=\mathrm{BAX}_{\mathrm{total}}-\mathrm{BAX}_{\mathrm{Cyto}}=749853 particles$ (16)

Mitochondrial BAX was distributed randomly across all mitochondria in proportion to their surface area. The translocation rate of BAX from cytosol to mitochondria was also derived from live-cell imaging data of YFP-BAX (Figure 5D in [11]). For each mitochondrion, basal BAX levels were assigned based on the whole cell BAX translocation simulations. Further BAX translocation was simulated by stepwise introducing cytosolic BAX to individual mitochondria based on the derived translocation rate. New BAX particles were seeded randomly across mitochondria, proportional to surface area, and added every minute over a 30-minute simulation with particle numbers adjusted according to the total BAX levels per mitochondrion. Retrotranslocation was modeled by removing inactive BAX particles (incorporating both unreacted and MCL1-mediated deactivated BAX) after each one-minute interval, thereby capturing BAX dynamics between cytosol and mitochondria. Reaction kinetics for BAX with other reactants were identical to those used for BAK (**Supp. Fig. 1E, F;** Material and Methods). This implementation enabled the simulation of protein reactions of the reduced MCL-1/tBID/BAX interactome with dynamic changes in mitochondrial BAX at single-mitochondrion resolution.

**Cell lines and antibodies**

NCI-H460 cells were obtained from ATCC (LGC Standards GmbH, Germany), HCT116 cells from Interlab Cell Line Collection (Italy) and HeLa cells from ATCC and cultured as previously described (11). Western blots and subsequent densitometry were performed as previously described (8,11) and were compared to HeLa reference cells as described in (12). The following antibodies were used: rabbit anti-Mcl-1 (D2W9E, Cell Signaling #94296, 1:1000 WB, 1:400 IF), rabbit anti-Bak (Cell Signaling #3814, 1:1000), rabbit anti-Bax (Cell Signaling #2772, 1:1000), goat anti-rabbit IgG (H+L) secondary antibody, Alexa Fluor 647 (Thermo Fisher Scientific, #A-21244, 1:500 IF) and peroxidase-conjugated goat anti-rabbit/mouse IgG (Dianova #111-035-144/115-035-062, 1:10,000).

**Supplemental references**

1. Kuritz K, Stöhr D, Maichl DS, Pollak N, Rehm M, Allgöwer F. Reconstructing temporal and spatial dynamics from single-cell pseudotime using prior knowledge of real scale cell densities. Sci Rep. 2020 Feb 27;10(1):3619.

2. Fullstone G, Bauer TL, Guttà C, Salvucci M, Prehn JHM, Rehm M. The apoptosome molecular timer synergises with XIAP to suppress apoptosis execution and contributes to prognosticating survival in colorectal cancer. Cell Death Differ. 2020 Oct;27(10):2828–42.

3. Rehm M, Huber HJ, Hellwig CT, Anguissola S, Dussmann H, Prehn JHM. Dynamics of outer mitochondrial membrane permeabilization during apoptosis. Cell Death Differ. 2009 Apr;16(4):613–23.

4. Potma EO, Boeij WP de, Bosgraaf L, Roelofs J, Haastert PJM van, Wiersma DA. Reduced Protein Diffusion Rate by Cytoskeleton in Vegetative and Polarized Dictyostelium Cells. Biophysical Journal. 2001 Oct 1;81(4):2010–9.

5. García-Sáez AJ, Ries J, Orzáez M, Pérez-Payà E, Schwille P. Membrane promotes tBID interaction with BCLXL. Nat Struct Mol Biol. 2009 Nov;16(11):1178–85.

6. Saffman PG, Delbrück M. Brownian motion in biological membranes. Proc Natl Acad Sci U S A. 1975 Aug;72(8):3111–3.

7. Hantusch A, Das KK, García-Sáez AJ, Brunner T, Rehm M. Bax retrotranslocation potentiates Bcl-xL’s antiapoptotic activity and is essential for switch-like transitions between MOMP competency and resistance. Cell Death Dis. 2018 Mar 22;9(4):1–13.

8. Pollak N, Lindner A, Imig D, Kuritz K, Fritze JS, Decker L, et al. Cell cycle progression and transmitotic apoptosis resistance promote escape from extrinsic apoptosis. Journal of Cell Science. 2021 Dec 16;134(24):jcs258966.

9. Todt F, Cakir Z, Reichenbach F, Emschermann F, Lauterwasser J, Kaiser A, et al. Differential retrotranslocation of mitochondrial Bax and Bak. The EMBO Journal. 2015 Jan 2;34(1):67–80.

10. Subburaj Y, Cosentino K, Axmann M, Pedrueza-Villalmanzo E, Hermann E, Bleicken S, et al. Bax monomers form dimer units in the membrane that further self-assemble into multiple oligomeric species. Nat Commun. 2015 Aug 14;6(1):8042.

11. Rehm M, Huber HJ, Dussmann H, Prehn JH. Systems analysis of effector caspase activation and its control by X‐linked inhibitor of apoptosis protein. The EMBO Journal. 2006 Sept 20;25(18):4338–49.

12. Lindner AU, Concannon CG, Boukes GJ, Cannon MD, Llambi F, Ryan D, et al. Systems Analysis of BCL2 Protein Family Interactions Establishes a Model to Predict Responses to Chemotherapy. Cancer Research. 2013 Jan 16;73(2):519–28.

**
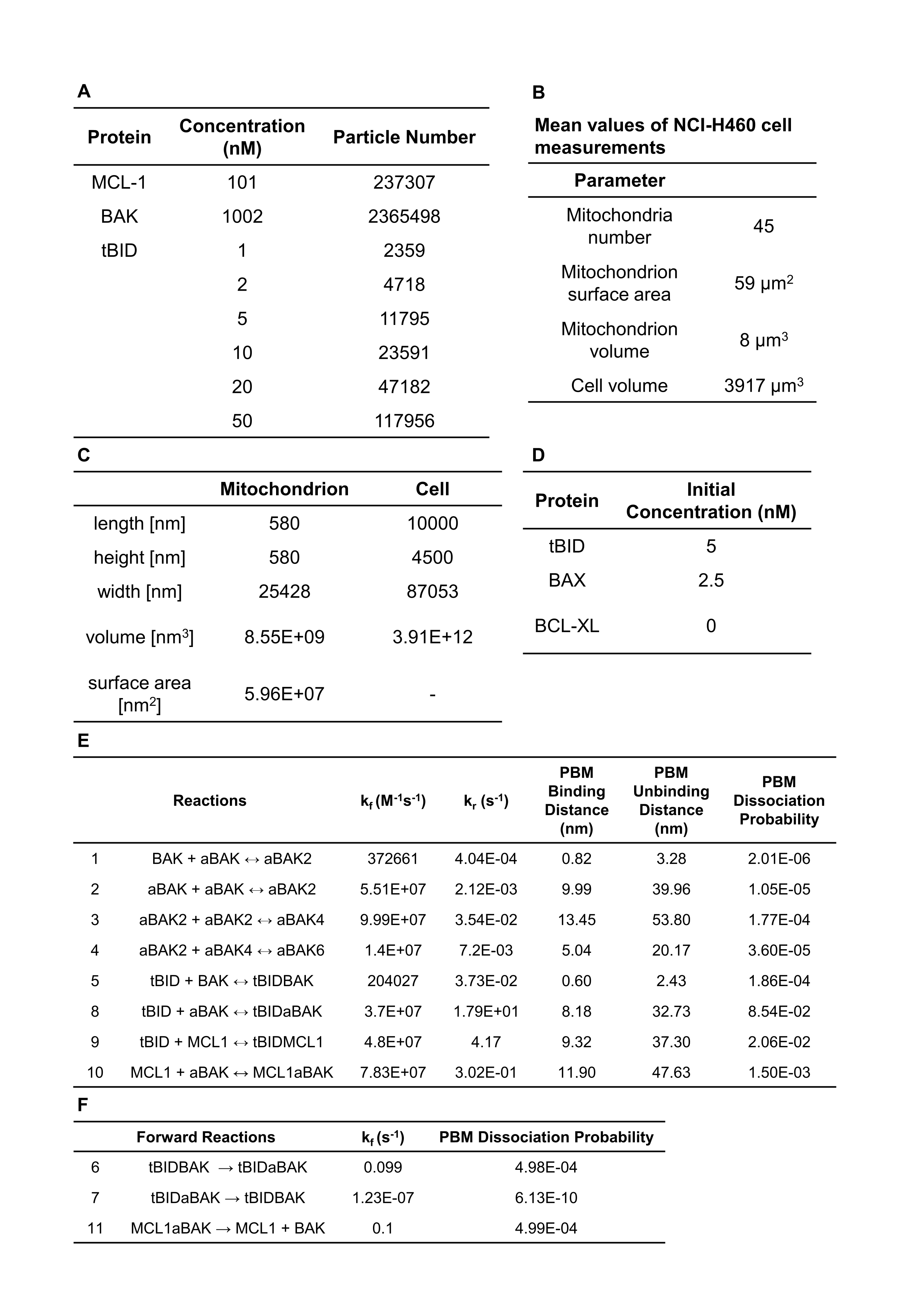
**

**Supp. Figure 1: Experimental data and parameterization for constructing a particle-based model of a mitochondrial network in a model cell.**

(A) Protein concentrations of selected BCL-2 family members in NCI-H460 cells.

(B) Mean morphological parameters derived from untreated NCI-H460 cells used for constructing a simplified model cell.

(C) Mitochondrial and cellular dimensions in the model cell were defined to match the experimentally derived measurements shown in (B). Mitochondria were represented as rectangular compartments with dimensions approximating the experimentally observed shapes.

(D) Initial concentrations for simulation of BAX-oligomer formation. Initial values were taken from (10).

(E) Protein interactions and corresponding reaction parameters of second order reactions. The full parameterization strategy is described in detail in the Methods section.

(F) Protein interactions and corresponding reaction parameters of forward reactions. The full parameterization strategy is described in detail in the Methods section.

**
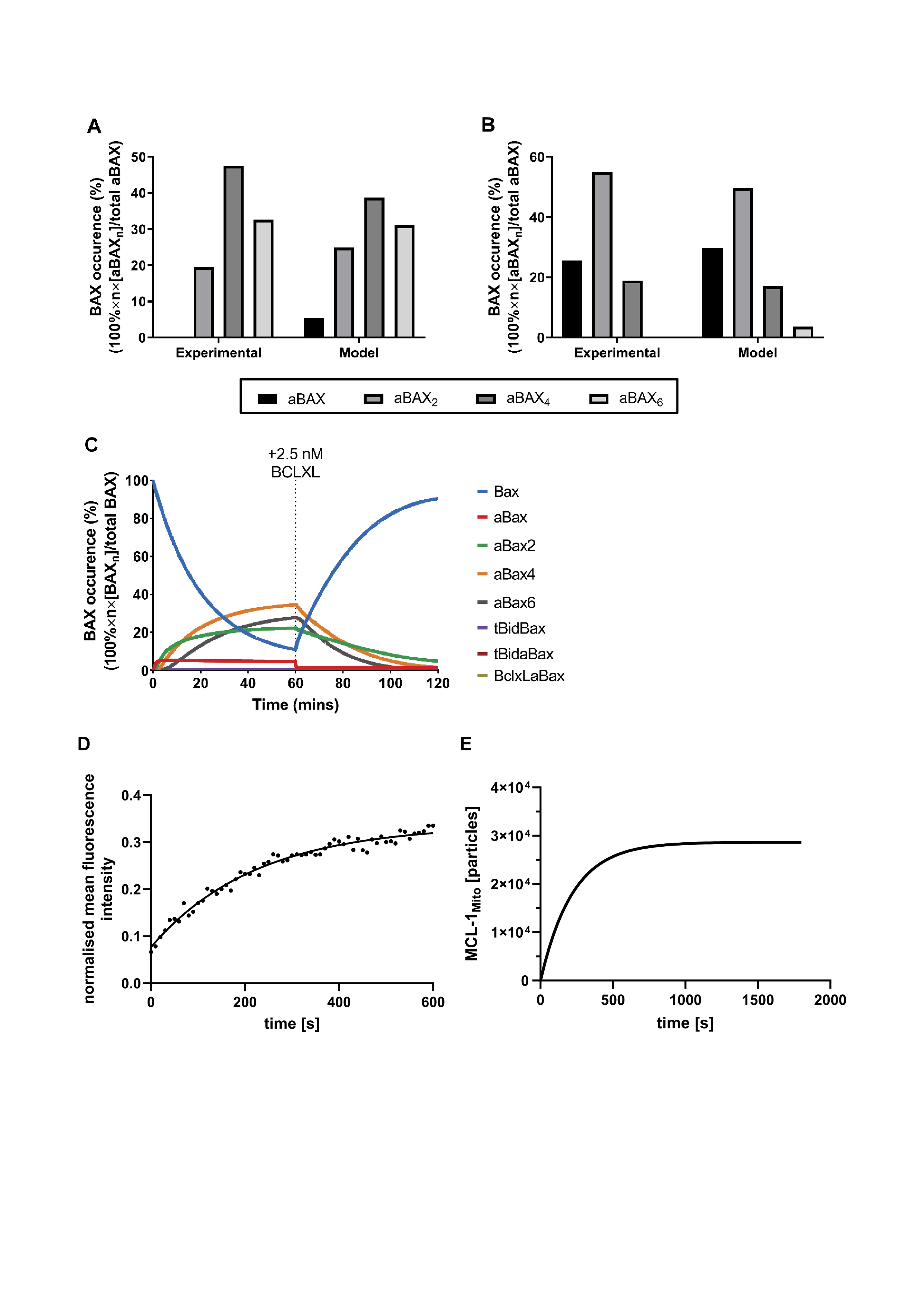
**

**Supp. Figure 2: (A-C) Replication of experimental kinetics and dynamics of BAX oligomerization.**

(A)-(C) Comparison of model and experimental data for Estimation Data 1 (A) and Estimation Data 2 (B). Experimental datapoints were extracted from Figure 3G (A) and 4D (B) in (10).

(C) Time-course dynamics of BAX oligomerization representing Experiment 1 (before addition of BCL-XL) and Experiment 2 (after addition of BCL-XL).

**Sup. Figure 2: (D-E) Experimental kinetic data for subcellular localization of MCL-1.**

(D) Normalized mean fluorescence intensities from FRAP experiments with MCL-1.
(E) Fluorescence recovery was fitted using a non-linear one-phase association model, yielding a rate constant of 0.00452 s⁻¹. Details of how this data were used to define MCL-1 localization dynamics in the model are provided in the Methods section.

**
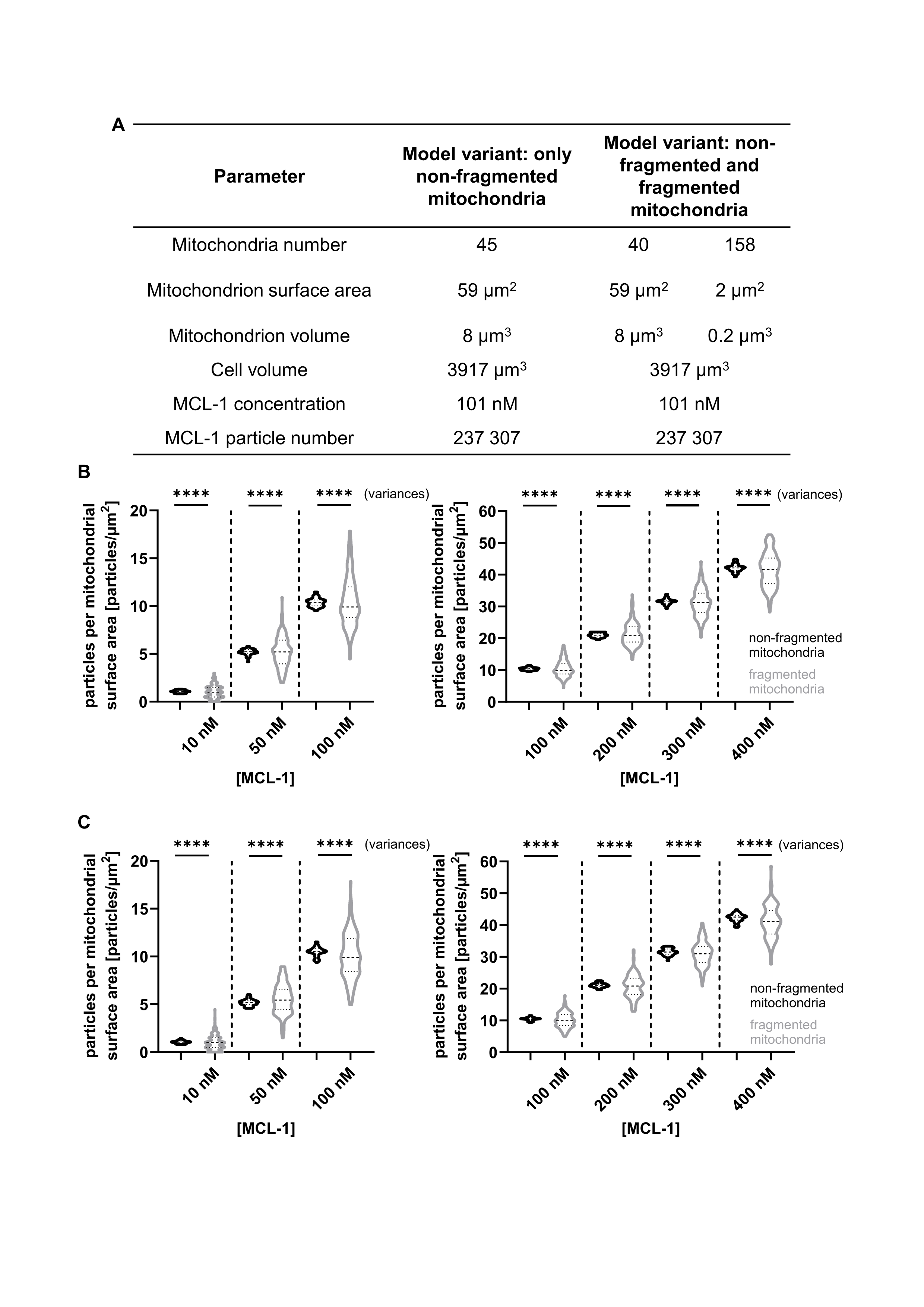
**

**Supp. Figure 3: Control simulations and model parameters for mitochondrial fragmentation.**

(A) Overview of model cell and mitochondrial parameters used for simulations involving mitochondrial fragmentation (see Fig. 3A). Cell size and total mitochondrial volume were kept constant across all conditions.

(B) Second independent simulation run of Fig. 3D. Median values did not differ significantly (Kruskal-Wallis, p > 0.05), but variance was consistently higher in fragmented mitochondria (Brown-Forsythe, p < 0.0001 across all concentrations).

(C) Third independent simulation run of Fig. 3D. As in (B), variance was significantly increased in fragmented mitochondria at all concentrations (Brown-Forsythe, p < 0.0001), while median values remained unchanged (Kruskal-Wallis, p > 0.05).

**
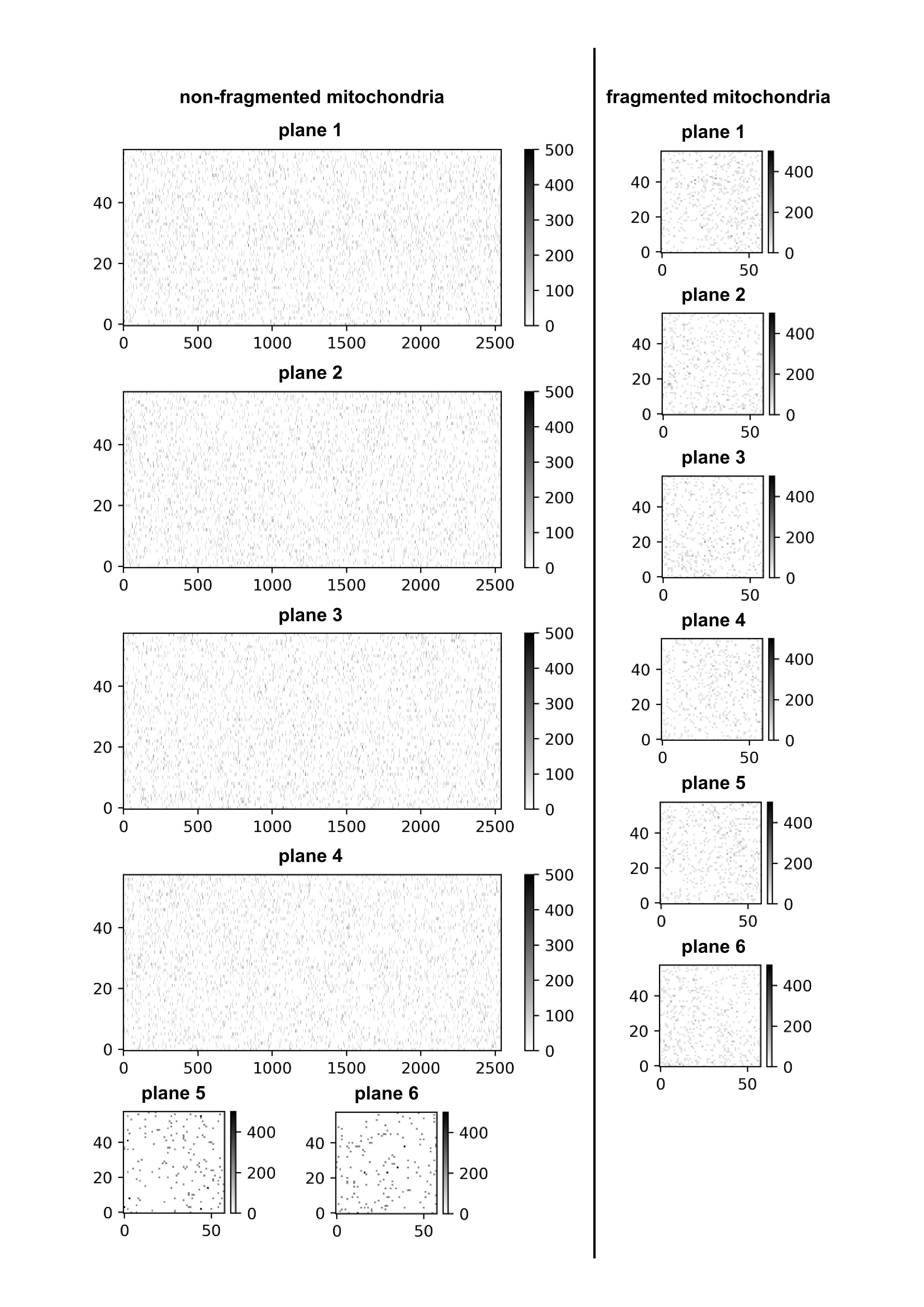
**

**Supp. Figure 4: Visualization of homogenous particle distributions across mitochondrial surface planes.**

Particle distributions were visualized using Matplotlib in combination with NumPy, displaying particle concentrations as intensity values across the mitochondrial surface plane. To analyse spatial distributions, each mitochondrial surface was divided into bins of 10 nm in length to capture local concentration differences with high spatial resolution.

**
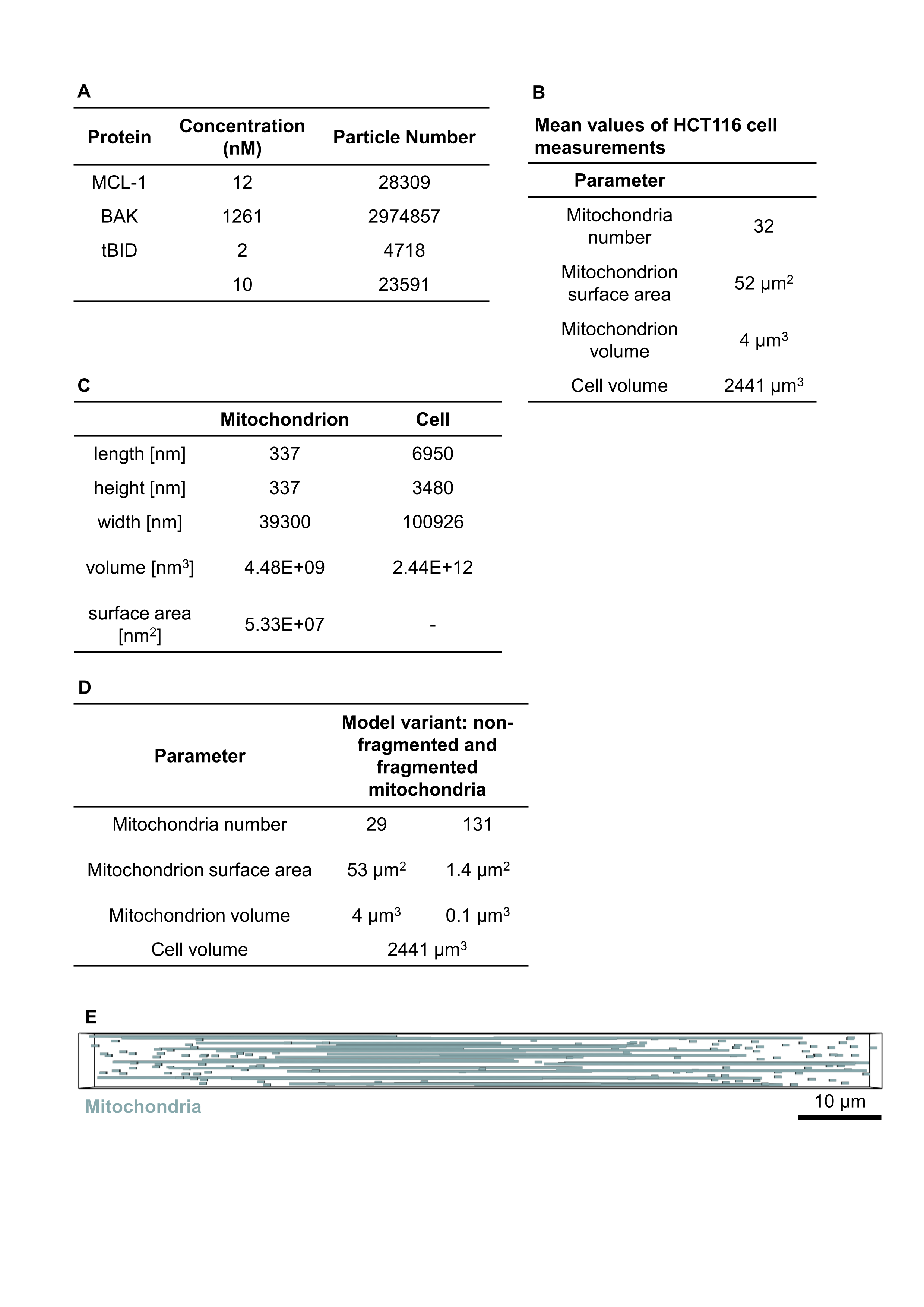
**

**Supp. Figure 5: Constructing a particle-based model of an HCT116 cancer cell.** (A) Protein concentrations of selected BCL-2 family members in HCT116 cells.

(B) Mean morphological parameters derived from untreated HCT116 used for constructing a simplified model cell.

(C) Mitochondrial and cellular dimensions in the model cell were defined to match the experimentally derived measurements shown in (B). Mitochondria were represented as rectangular compartments with dimensions approximating the experimentally observed shapes.

(D) Overview of model cell and mitochondrial parameters used for simulations involving mitochondrial fragmentation. Cell size and total mitochondrial volume were kept constant across all conditions.

(E) Visualization of the particle-based model with implementation of mitochondrial fragmentation (10% of mitochondria). Total mitochondrial volume was kept constant. Mitochondria are shown in blue; particles are hidden for clarity.

**
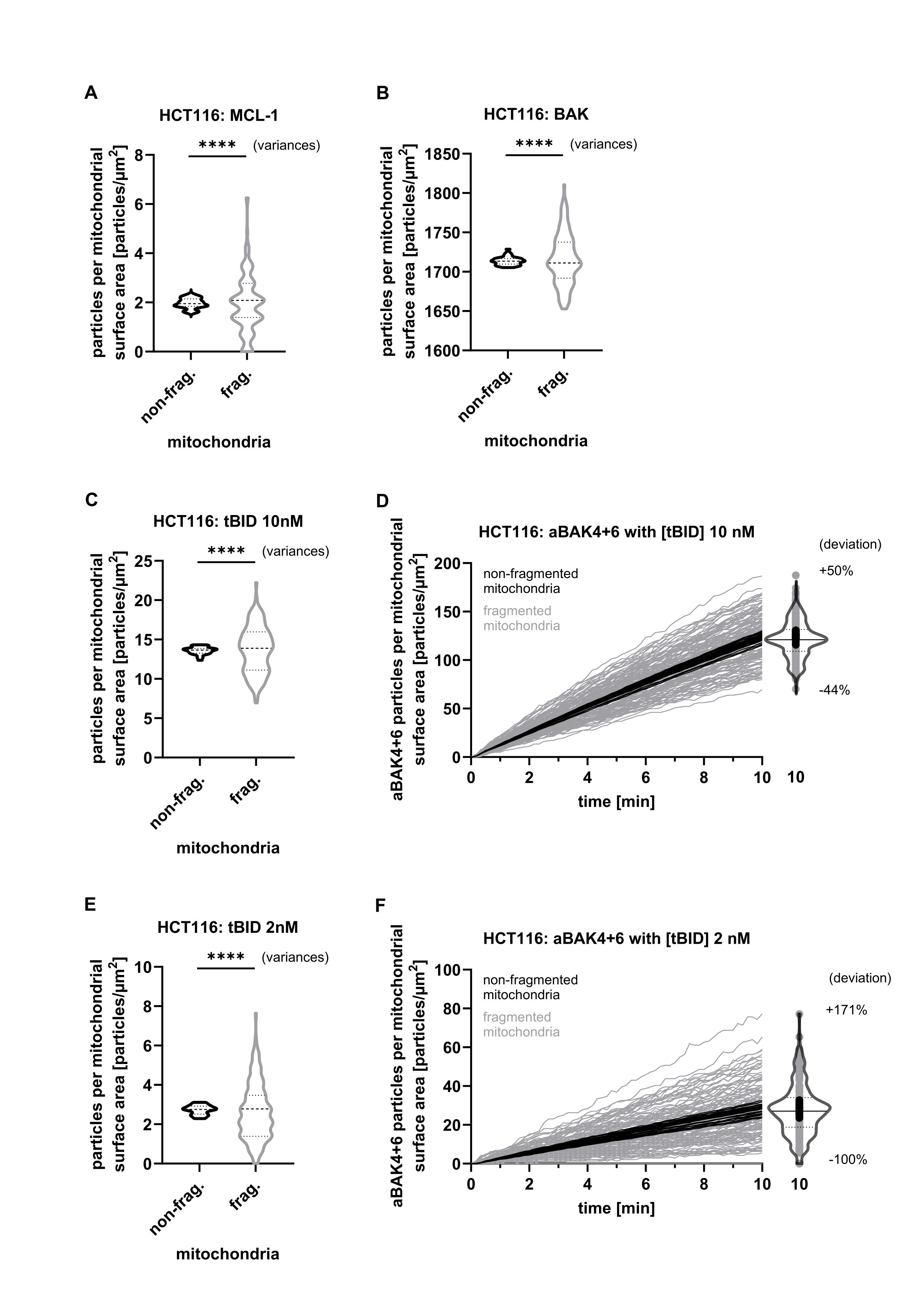
**

**Supp. Figure 6: Mitochondrial fragmentation increases the variability of mitochondrial-bound proteins and MOMP susceptibility in HCT116 cells.**

(A-C) Quantification of protein densities for MCL-1 (A), BAK (B), and 10 nM tBID (C) in HCT116 cells, at the steady-state (0 min) prior to simulating protein interactions. Medians comparison by Mann-Whitney U test (p > 0.05). Variances comparison by Levene’s test (p < 0.0001).

(D) Formation of BAK pore complexes (aBAK4+6) was simulated over time with 10 nM tBID. Each line represents a single mitochondrion. Results from fragmented (grey) and non-fragmented (black) mitochondria are shown. End-point distributions are visualized as violin and scatter plots.

(E) Steady-state quantification of 2 nM tBID associated with mitochondria before simulating protein interactions. Medians comparison by Mann-Whitney U test (p > 0.05). Variances comparison by Levene’s test (p < 0.0001).

(F) Formation of BAK pore complexes (aBAK4+6) was simulated over time with 2 nM tBID. Each line represents a single mitochondrion. Results from fragmented (grey) and non-fragmented (black) mitochondria are shown. End-point distributions are visualized as violin and scatter plots.

**
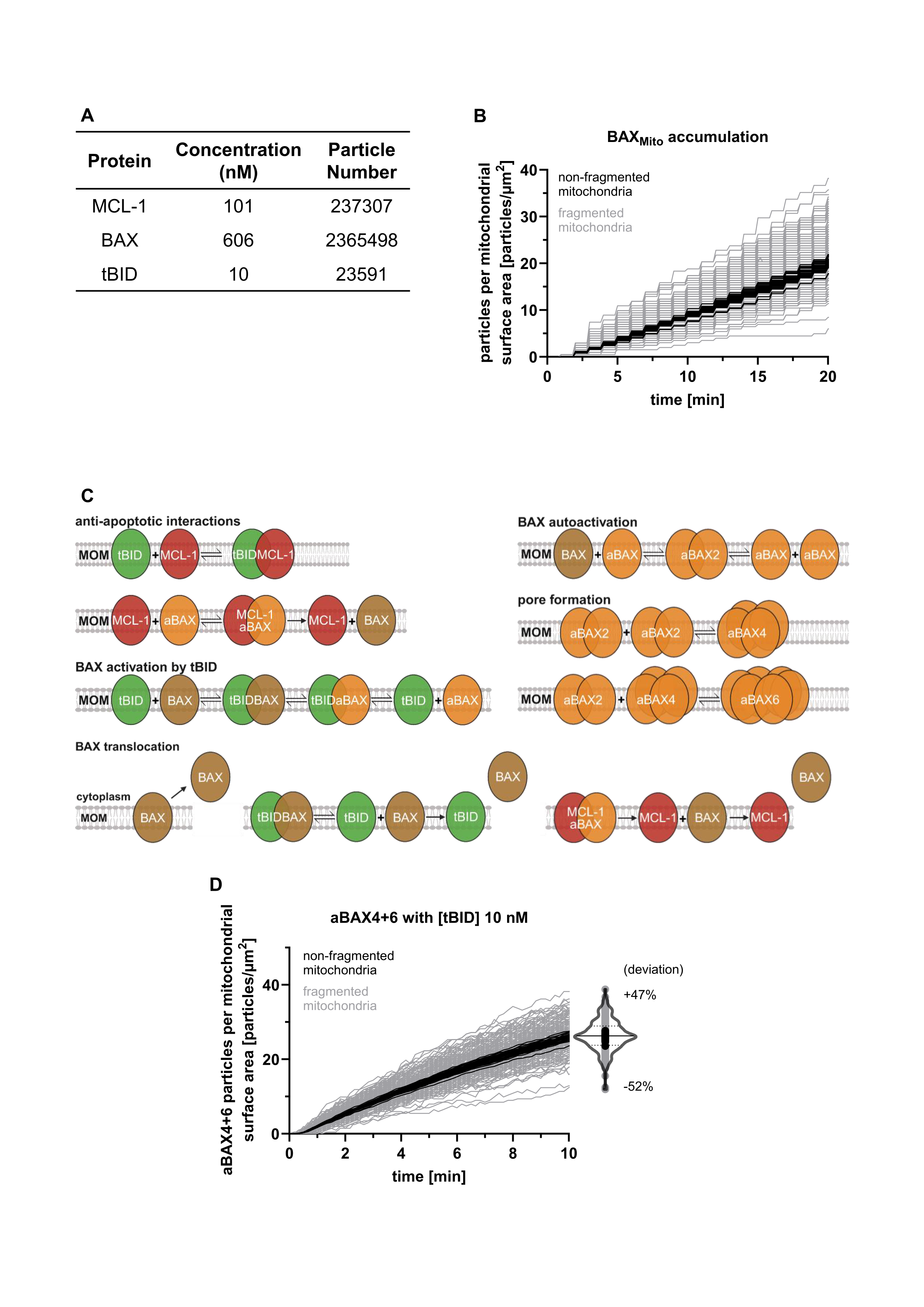
**

**Supp. Figure 7:** **Heterogenous MOMP susceptibility is maintained in models incorporating the translocation and activation of BAX.**

(A) Protein concentrations of selected BCL-2 family members in NCI-H460 cells.

(B) BAX accumulation at the mitochondria due to active apoptosis signalling. Data represents total mitochondrial BAX in the presence of 10 nM tBID subtracted by the same levels in the absence of tBID. Each line represents a single mitochondrion. Results from fragmented (grey) and non-fragmented (black) mitochondria are shown.

(C) Schematic of the implemented mitochondrial interaction network showing anti-apoptotic MCL-1 (red), pro-apoptotic tBID (green), and BAX in inactive (brown) and active (orange) states.

(D) Formation of BAX pore complexes (aBAX4+6) was simulated over time with 10 nM tBID. Each line represents a single mitochondrion. Results from fragmented (grey) and non-fragmented (black) mitochondria are shown. End-point distributions are visualized as violin and scatter plots.
